# High expression of small GTPase Rab3D promotes cancer progression and metastasis

**DOI:** 10.18632/oncotarget.3575

**Published:** 2015-03-14

**Authors:** Jian Yang, Wei Liu, Xin'an Lu, Yan Fu, Lin Li, Yongzhang Luo

**Affiliations:** ^1^ National Engineering Laboratory for Anti-tumor Protein Therapeutics, Tsinghua University, Beijing, China; ^2^ Beijing Key Laboratory for Protein Therapeutics, Tsinghua University, Beijing, China; ^3^ Cancer Biology Laboratory, School of Life Sciences, Tsinghua University, Beijing, China

**Keywords:** Rab3D, EMT, exosomes, Hsp90α, tumor metastasis

## Abstract

Rab GTPases control exocytic and endocytic membrane trafficking such as exosomes release. As a secretory small GTPase, Rab3D is a vital regulator for protein secretion. However, the role of Rab3D in cancer was never systematically studied. The aim of this study is to examine its function and mechanism in cancer, especially metastasis. We detected protein levels of Rab3D in nine cancer cell lines and twelve types of clinical cancer specimens. Subsequently, we established *in vitro* migration and *in vivo* orthotopic metastatic mouse models to study the role of Rab3D in tumor metastasis. Here, we reported that the expression levels of Rab3D were dysregulated in cancer cells and highly correlated with tumor malignancies in the clinical samples. Increased expressions of Rab3D led to tumor invasion *in vitro* and lung metastasis *in vivo*, whereas Rab3D knockdown suppressed the tumor cell motility. Mechanistic studies revealed that Rab3D activated intracellular the AKT/GSK3β signaling to induce the EMT process. In addition, it also regulated the extracellular secretion of Hsp90α to promote tumor cell migration and invasion. These results prove that Rab3D is a key molecule to regulate tumor metastasis, suggesting that blocking the Rab3D function can be a potential therapeutic approach for cancer metastasis.

## INTRODUCTION

More than 90% of cancer mortality is attributable to metastases, thus the prevention of metastasis guides the development of novel diagnostic and therapeutic strategies [1]. In the tumor microenvironment, cell migration and invasion are regulated by many factors such as intracellular signaling pathways, growth factors or onco-proteins [2]. In recent years, increasing numbers of studies have revealed that vesicular exocytosis can also play an essential role in tumor progression and malignancy [3].

Exosomes, characterized by a size of 30-100 nm in diameter, have been viewed as secreted vesicles that enable extracellular communications [4-6] and have played an important role in tumor progression and metastasis by promoting the extracellular matrix degradation and also by remodeling to establish the metastasis niche [7, 8]. Abundant proteins, including membrane trafficking molecules, cytoskeleton molecules, chaperones, signal transduction proteins and cytoplasmic enzymes have been included in secreted exosomes [9].

Rab GTPases, as intracellular transport proteins, are master regulators of exocytic and endocytic membrane trafficking [10]. The different secretory processes are controlled by corresponding Rab proteins including Rab3A/B/C/D, Rab26, Rab27A/B and Rab37. Aberrant expressions of Rabs can influence cancer development and metastasis [11]. Kim and coworkers have shown that Rab3A is a novel oncogene involved in glioma initiation and progression *via* promoting cell proliferation [12]. Another Rab3 subfamily member Rab3B has been reported to be overexpressed in prostate cancer patients, which reduces tumor cell death [13]. Rab37 acts as a metastasis-related tumor suppressor gene in lung cancer, and low mRNA expression of Rab37 is significantly associated with lung metastasis [14]. Besides, Rab27 has been reported to control essential steps of the exosomes secretion pathway [15]. Rab27A promotes tumor progression by mediating the secretion of cytokines and exosomes in tumor microenvironment [16]. At the meantime, high level of Rab27B has been found in a poor prognostic phenotype of human breast cancer, which is due to the regulatory effect on invasive growth and metastasis [17-19]. In Rab27 regulated exosomes, the indispensable and ubiquitous molecular chaperone Hsp90α has been found to enhance cancer cell invasion through activating matrix metalloproteinases (MMPs) [17, 20]. Hsp90α, an isoform of the Hsp90 family, can be translocated to the cell surface [21] and secreted into the extracellular space [22, 23]. Our previous studies have demonstrated that the level of plasma Hsp90α is positively correlated with the tumor malignancy in clinical cancer patients and the secreted Hsp90α stabilizes MMP-2 to facilitate tumor invasion [24, 25]. These above reports suggest that the abnormal expression of secretory Rab GTPases could be a generalized feature of tumor progression.

Rab3D is another very important member of the secretory Rab GTPases. Comparing to Rab26 and Rab37, which modulate the secretion in specialized cell types, Rab3D is enriched in non-neuronal tissues, whereas Rab3A/B/C are mostly expressed in the nervous system. In addition, the N- and C-terminal regions of Rab3D are largely distinct from other Rab3 isoforms. Rab3D regulates exocytosis processes and apically directs transcytosis [26-28]. Although Rab3D is widely expressed in several secretory tissues and even in rat pancreatic acinar tumor cell line AR42J cells [29], the biological function and molecular mechanisms for Rab3D in cancer have not been elucidated so far.

Here, we demonstrated that Rab3D promotes breast cancer cell invasion and lung metastasis by EMT induction through the activation of the AKT/GSK-3β/Snail signaling pathway. More importantly, a significant positive relationship between Rab3D and the malignancy of cancer patients is observed. Thus, this study provides a novel insight into the biomedical relevance of Rab3D in tumor malignancies, which indicates that Rab3D plays a critical role in promoting tumor metastasis and is a promising therapeutic target for the treatment of cancer.

## RESULTS

### Rab3D is aberrantly elevated in human cancers and correlated with the malignancy

**Figure 1:**
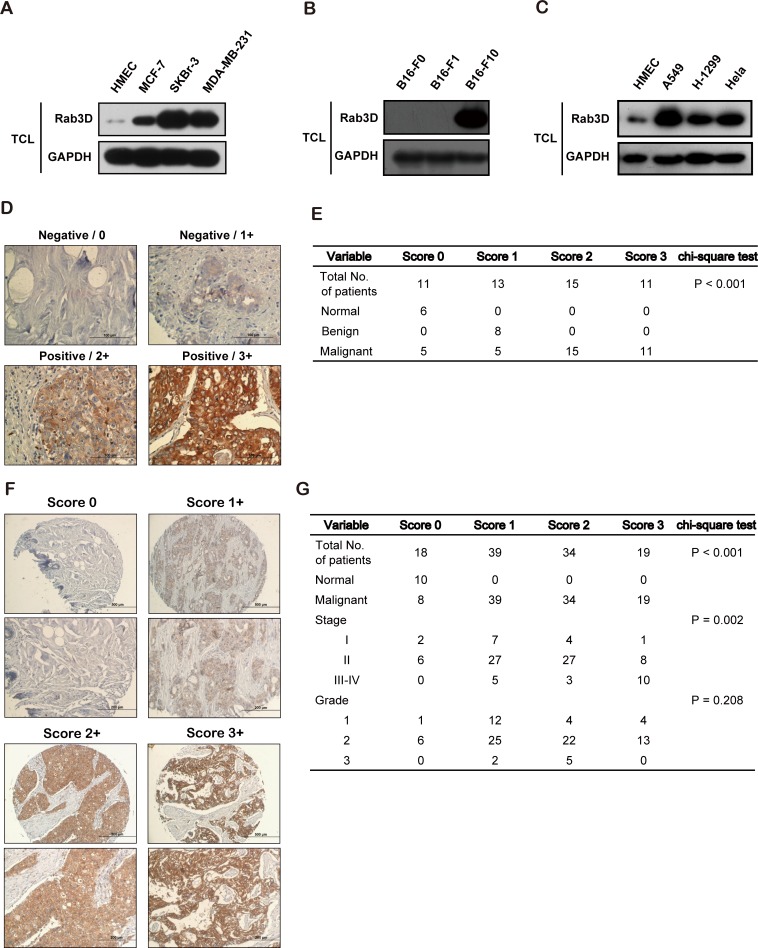
Increased Rab3D in malignant tumor cells and in clinical cancer specimens (A). Western Blot analysis of intracellular Rab3D expression in HMEC, non-invasive breast cancer MCF-7, and invasive SKBr-3, MDA-MB-231 cell lines. (B). Western Blot analysis of intracellular Rab3D expression in melanoma F0, F1, F10 cell lines. (C) Western Blot analysis of intracellular Rab3D expression in lung cancer A549, H-1299 cell lines and ovary cancer Hela cell line. (D). Representative Rab3D staining in normal tissues, benign tumor and malignant breast cancer samples that illustrate immunohistochemical scores of 0, 1, 2 and 3. Scale bar, 100 μm. (E). Association of Rab3D immunohistochemical scores with tumor malignancy. The *x*^2^ test was used to test difference between categorical variables. (F). Representative Rab3D staining in different stages of malignant breast cancer samples that illustrate immunohistochemical scores of 0, 1, 2 and 3. Scale bar, 100 μm. (G). Associations of Rab3D immunohistochemical scores with tumor stage (I, II, III-IV) and tumor grade (1, 2, and 3). The x^2^ test was used to test difference between categorical variables.

To explore the role of Rab3D in tumor progression, we firstly analyzed the level of Rab3D in cancer cells. The levels of intracellular Rab3D in many types of cancer cells were significantly higher than that in the immortalized human microvascular endothelial cells (HMEC) (Fig. 1A-C). In addition, in breast cancer cells with different malignancies, the levels of intracellular Rab3D were highly elevated in invasive SKBr-3 and MDA-MB-231 cell lines compared with that in the non-invasive MCF-7 (Fig. 1A), showing a positive correlation between breast cancer cell aggressive phenotypes and Rab3D expression levels. Also in melanoma cell lines, the levels of intracellular Rab3D were also aberrantly up-regulated in more malignant F10 cancer cells (Fig. 1B). And in other type of cancer cells such as lung cancer, the high expression of Rab3D was also observed (Fig. 1C).

Next, we examined the Rab3D expression in clinical cancer patients' samples using immunohistochemistry on a breast cancer tissue microarray containing 50 specimens. Tissues were scored on the basis of staining intensities of Rab3D expressions and the percentages of cancer cells stained. We found that 72.2 % (26/36) malignant breast cancer tissues showed the positive staining, whereas the intensity of Rab3D staining in normal tissues or benign breast tumors was negative (Fig. 1D-E, Fig. S1A and Table S1). More intriguingly, similar to breast cancer tissue, high expression levels of Rab3D were also observed in other types of cancers including prostate, lung, colon, ovary, liver, uterine cervix, esophagus and skin carcinoma tissues (Fig. S1C and Table S2), indicating that Rab3D may play an important and critical role in the progression of these tumor types.

To further confirm the clinical relevance that Rab3D is positively correlated with tumor malignancy, we detected the level of Rab3D using another breast tissue microarray containing 110 cases with known clinical records and analyzed Rab3D's association with clinical and pathological parameters including staging and grading. Notably, IHC staining showed that Rab3D was markedly increased in stage III-IV (*P*=0.002), but not related to tumor grade (*P*=0.208), showing a significant correlation between Rab3D expressions and cancer TNM stages (Fig. 1F-G, Fig. S1B and Table S3).

### Rab3D regulates cancer cell motility and invasiveness

To investigate whether Rab3D is required for the invasive phenotypes of cancer cells, we knocked down Rab3D in highly invasive MDA-MB-231 cells and highly expressed Rab3D in non-invasive MCF-7 cells, respectively. The Western Blot analysis proved the efficiencies of siRNA-mediated Rab3D knockdown and its overexpression (Fig. S2A-B).

**Figure 2:**
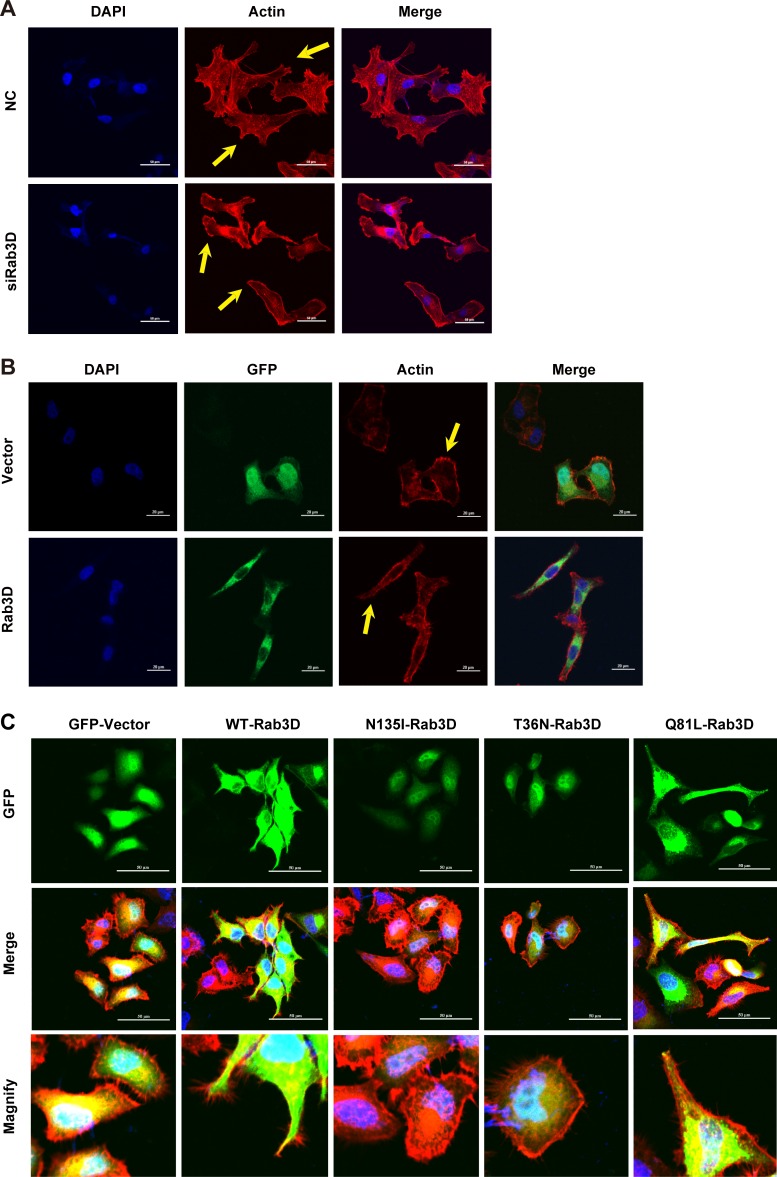
The effect of Rab3D on tumor cells morphology and ruffle formation (A). The effect of Rab3D on cytoskeletal rearrangements. Control and siRab3D MDA-MB-231 cells were kept in serum and then fixed. Membrane ruffles formation was analyzed by confocal microscope using a Rhodamine labeled phalloidin antibody to visualize actin cytoskeleton. Scale bar, 50 μm. (B). The effect of overexpressing Rab3D on tumor cell morphology. MCF-7 cells transiently transfected with GFP or GFP-Rab3D-expressing plasmid. Nuclei were stained with DAPI and actin was stained with Rhodamine labeled phalloidin antibody. (C). The effect of different Rab3D mutants on tumor cell morphology. Rab3D (Green), Actin (Red), DAPI (Blue). Scare: 50 μm.

Cell motility driven by cytoskeletal rearrangement plays an essential role in the development of metastasis [30]. We thus explored the effect of Rab3D on actin cytoskeleton. As shown in Fig. 2A and Fig. S2C, Rab3D knockdown clearly inhibited the membrane ruffling formation and shrank the elongated morphology. Furthermore, we found that MCF-7 cells transfected with GFP-Rab3D formed the cellular extension and spreading protrusion, whereas the GFP control group exhibited no morphological change (Fig. 2B and S2D). Rab3D is a small GTP-binding protein and acts as the molecular switch between the GDP-bound inactive and GTP-bound active forms. We therefore constructed three mutations of Rab3D. N135I-Rab3D and T36N-Rab3D are both dominant negative mutants which are defective in the GTP binding. Q81L-Rab3D is a constitutively active mutant defective in the GTP hydrolysis [31]. MCF-7 cells transfected with N135I-Rab3D and T36N-Rab3D did not change their morphologies, while Q81L-Rab3D-expressing cells showed elongated protrusions (Fig. 2C), indicating that Rab3D enhances tumor cell motility depending on its GTP binding activity.

**Figure 3:**
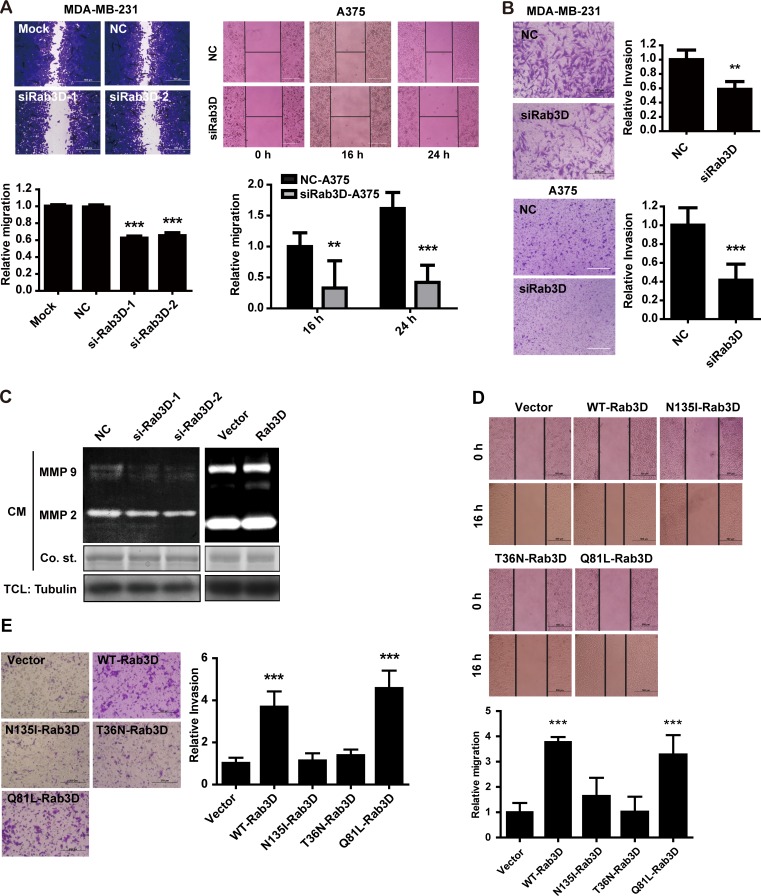
The effect of Rab3D on tumor cell migration and invasion *in vitro* (A). Down-regulation of Rab3D markedly repressed tumor cells migration (wound healing assays). Representative images and quantification of migration assay in Rab3D knockdown MDA-MB-231 cells (Scale bar, 200 μm) or A375 cells (Scale bar, 50 μm). ** *p* < 0.01, *** *p* < 0.001. (B). Down-regulation of Rab3D inhibited the invasion capability of tumor cells on Matrigel-coated transwell insets in the invasion assay. Representative images and quantification of invasion assay by MDA-MB-231 cells (Scale bar, 100 μm) or A375 cells (Scale bar, 50 μm) in which Rab3D was depleted. ** *p* < 0.01, *** *p* < 0.001. (C). The effect of Rab3D on MMP-2 and MMP-9 activity. Gelatin zymography analysis of MMP-2 and MMP-9 activity in CM from Rab3D knockdown MDA-MB-231 cells or Rab3D overexpressing MCF-7 cells. (D). Representative images and quantification of migration assay by MCF-7 cells transfected to express Rab3D mutation. Scale bar, 200 μm. *** *p* < 0.001. (E). Representative images and quantification of the invasive phenotype. Scale bar, 100 μm. ***p < 0.001.

To ascertain the role of Rab3D in tumor motility, wound healing assays were carried out. The migration abilities were markedly repressed in MDA-MB-231 cells transfected with Rab3D siRNA (Fig. 3A). Similar inhibition effects were obtained when detecting the invasion capabilities of tumor cells on Matrigel-coated transwell inserts in the invasion assay (Fig. 3B). siRab3D knockdown-reduced migration and invasion were also observed in other cell line such as melanoma A375 cells (Fig. 3A and 3B). Matrix metalloproteinases (MMPs) have played important roles in the degradation of the extracellular matrix to promote tumor invasion and metastasis [32]. MMP-9 activity was significantly decreased when knocking down Rab3D (Fig. 3C). In contrast, the overexpression of Rab3D or Q81L-Rab3D in MCF-7 enhanced cell motility and invasiveness dramatically (Fig. 3D-E, Fig. S2E-G). Taken together, these results prove that Rab3D is indispensable for cell migration and invasion *in vitro* in a GTP dependent manner.

### Rab3D induces epithelial to mesenchymal transition

To unravel the molecular mechanisms by which Rab3D regulates cancer progression, we focused on the epithelial to mesenchymal transition (EMT) induction, because the process of EMT has been documented to be associated with tumor progression and metastasis [33].

**Figure 4:**
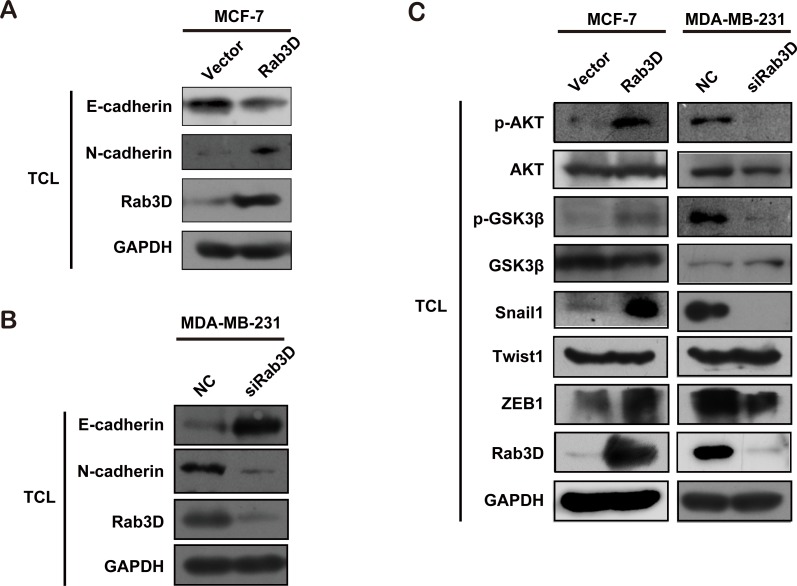
Rab3D as a regulator in EMT-like changes (A). Western Blot analysis in Rab3D MCF-7 cells of EMT marker (E-cadherin and N-cadherin). (B). Measurement of E-cadherin or N-cadherin expression in siRab3D MDA-MB-231 cells. (C). The EMT-related signaling activation (AKT/GSK3β/Snail) in different group.

It has been reported that the level of mesenchymal marker N-cadherin in MDA-MB-231 cells is highly elevated compared with MCF-7 cells [34]. To uncover whether Rab3D induces EMT process, we detected EMT marker in Rab3D-MCF-7 cells and siRab3D-MDA-MB-231 cells, respectively. As expected, Rab3D overexpression increased N-cadherin expression while decreased the level of epithelial marker E-cadherin (Fig. 4A). Conversely, Rab3D knockdown resulted in a decreased N-cadherin expression level, but increased the level of E-cadherin in siRab3D-MDA-MB-231 cells (Fig. 4B). Furthermore, the expression levels of phosphorylated AKT, phosphorylated GSK3β, ZEB-1 and snail-1, all of which are very important for EMT process during cancer progression, were significantly up regulated in Rab3D-MCF-7 cells, but decreased in siRab3D-MDA-MB-231 cells (Fig. 4C).

### Rab3D regulates Hsp90α secretion to promote tumor cells invasion

**Figure 5:**
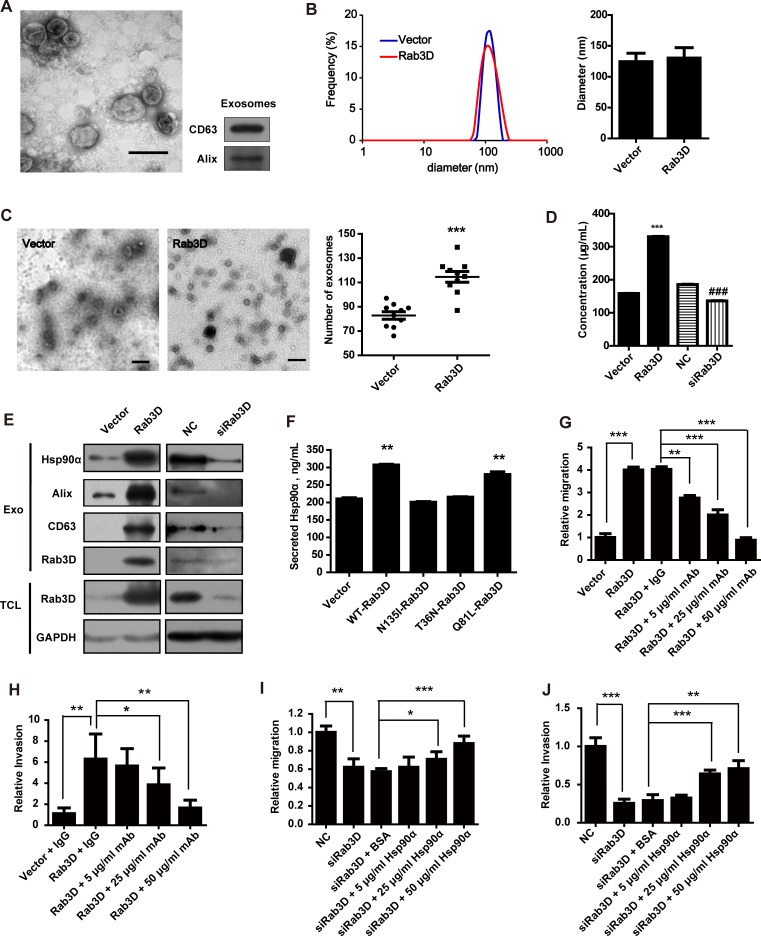
The role of secreted Hsp90α in Rab3D induced invasion (A). Identification and characterization of exosomes. Exosomes were isolated by sequential centrifugations from supernatants. Scale bar, 100 nm. An inset panel shows the exosomal marker, tetraspanin protein CD63 and Alix by Western Blot. (B). The size distribution of exosomes was analyzed by DLS and given as average ± standard deviation (n = 3). (C). Representative images and quantification of exosomes. Scale bar, 200 nm. *** *p* < 0.001. (D). The total protein level of extracellular exosomes was detected by BCA (n = 3). (E). Western Blot analysis of exosomal marker CD63 and Alix in exosomes from Rab3D-MCF-7 cells or siRab3D-MDA-MB-231 cells. (F) The level of secreted Hsp90α was detected by ELISA assay when the MCF-7 cells were transfected with different plasmid. (G). Quantification of migration assay of recombinant Hsp90α treated MDA-MB-231 transfected with scramble RNA or Rab3D siRNA. Statistically significant *p* values are indicated. ** *p* < 0.01, *** *p* < 0.001. (H). Measurement of invasion in siRab3D MDA-MB-231 with or without addition of recombinant Hsp90α. * *p* < 0.05, ** *p* < 0.01, *** *p* < 0.001. (I). Quantification of migration assay. MCF-7 cells that expressed Rab3D, with or without Hsp90α neutralizing antibody, were seeded. The relative migration distance was calculated after 16 h. * *p* < 0.05, ** *p* < 0.01, *** *p* < 0.001. (J). Quantification of matrigel invasion assay. The effect of blocking extracellular Hsp90α in MCF-7 cells over-expressing Rab3D on invasive ability. ** *p* < 0.01, *** *p* < 0.001.

To further investigate the mechanism how Rab3D promotes cells invasion, we firstly detected the effect of Rab3D on the release of exosomes. Extracellular exosomes were isolated by sequential centrifugation (Fig. 5A). The size range of exosomes and their average diameter were not changed after overexpressing Rab3D (Fig. 5B), whereas the number of released exosomes was significantly increased (Fig. 5C). In addition, Rab3D overexpression resulted in the elevated concentration of the exosomes protein extracts and the level of exosomal markers such as Alix and CD63, conversely, siRNA-mediated knockdown of Rab3D reversed this phenotype (Fig. 5D-E). These data suggest that Rab3D controls the release of exosomes.

Secreted Hsp90α plays an essential role in tumor invasion [24, 35]. Our group has shown that the level of plasma Hsp90α is positively correlated with tumor malignancy in patients samples [24]. Clinical trials with the enrollment of 2,347 cases validated plasma Hsp90α as a novel tumor biomarker. The ELISA kit for Hsp90α detection has been approved by China Food and Drug Administration (CFDA) and obtained the European Union CE and ISO13485 certifications, respectively. It has been reported that Hsp90α exists in exosomes and its secretion can be modulated by Rab27 [17, 20], we thus wondered whether Rab3D also regulate Hsp90α secretion.

Rab3D can dramatically facilitate the secretion of Hsp90α compared with other Rabs such as Rab11A, Rab11B, Rab27A, Rab27B, Rab35 and Rab37 (Fig. S3H and S3I). Moreover, the knockdown of Rab3D in MDA-MB-231 cells could reduce Hsp90α secretion compared with the control cells (Fig. S3A). Western Blot and Mass Spectrometry analysis confirmed that Hsp90α was among the up-regulated proteins in the CM of Rab3D-MCF-7 cells (Fig. S3B and S3C). Besides, the secretion of Hsp90α is dependent on the GTPase function of Rab3D (Fig. 5F). These results demonstrated that Rab3D indeed regulated Hsp90α secretion.

Secreted Hsp90α is known to be essential in cancer cell invasion through stabilizing extracellular proteins such as MMPs or active intracellular signaling [36]. We found that the loss of migration and invasion by neutralization of secreted Hsp90α with its antibodies cannot be rescued by overexpression of Rab3D (Fig. 5G-5H and Fig. S3D-E). In accordance, the abrogation of the decreased migration and invasion in the cells treated with siRNA of Rab3D was observed when adding the recombinant Hsp90α to the culture medium (Fig. 5I-5J and Fig. S3F-G). These results demonstrate that Rab3D promotes cancer cell migration and invasion *via* Hsp90α release.

### Rab3D is necessary for tumor metastasis and progression *in vivo*

**Figure 6:**
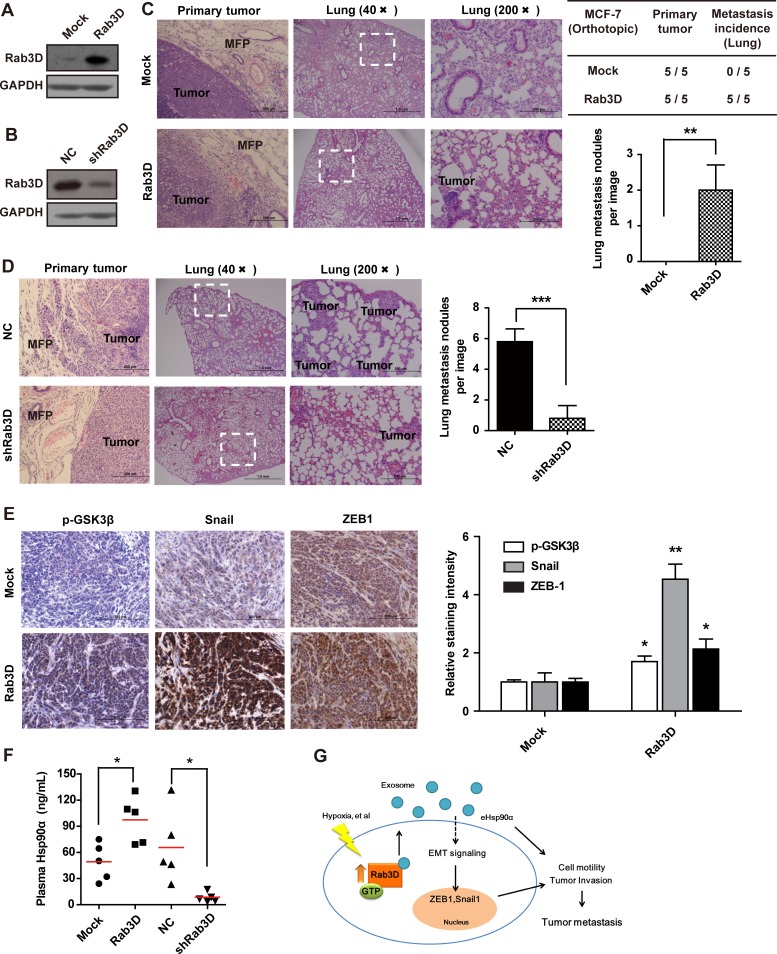
*In vivo* effects of Rab3D on tumor metastasis (A-B). Rab3D expression in stably transfected tumor cell lines. (C). Representative images of primary tumors and lung section stained with hematoxilin and eosin in control and Rab3D-MCF-7 xenografts bearing mice (n = 5 mice per group). (D). H & E staining of lung in control and shRab3D-MDA-MB-231 xenografts bearing mice. (E). Immunohistochemical staining images and quantification of EMT-related signaling activation in control and Rab3D-MCF-7 xenografts. Scale bar, 50μm. (F). The level of plasma Hsp90α in nude mice detected by ELISA assay (n = 5 mice per group). (G). The working model for Rab3D-induced tumor cell invasion. In response to hypoxia, intracellular Rab3D is increased and its expression is correlated with tumor malignancy. Rab3D regulates exosomes release and Hsp90α secretion, which promotes EMT and tumor metastasis. eHsp90α indicates extracellular Hsp90α.

To further test the significance of Rab3D in tumor progression, we next investigated the function of Rab3D in tumor metastasis and progression *in vivo*. At first, we established the cancer cell lines stably overexpressing recombinant human Rab3D (Fig. 6A and Fig. S4A) and stably transfecting with GFP-Rab3D shRNA, respectively (Fig. 6B and Fig. S4B). We injected these cells separately into mammary fat pads of nude mice to establish spontaneous metastasis model and monitored tumor growth and metastasis. Clear boarders without obvious invasion to surrounding tissues were observed in the control group, while tumors formed by Rab3D-MCF-7 cells invaded surrounding muscles, mammary fat pads as well as lung tissues (Fig. 6C and Fig. S4C), demonstrating that Rab3D-MCF-7 cells became more invasive. In the metastatic lung, we observed small metastatic clones with GFP density near the blood vessels in the Rab3D-MCF-7 group. Conversely, stable knockdown of Rab3D in xenograft tumor model is sufficient to reduce metastatic colonies in the lung (Fig. 6D and Fig. S4D). In addition, there were no obvious differences of cell proliferation *in vitro* (Fig. S5A and S5B) and tumor growth *in vivo* (Fig. S5C-F) among different groups, although the overexpression of Rab3D decreased apoptosis to some extent (Fig. S5G), demonstrating that Rab3D is highly specific for promoting tumor invasion and metastasis but not due to changes in proliferation.

Consistent with our observations *in vitro*, the levels of Rab3D expression were positively correlated with the levels of N-cadherin, phosphorylated GSK3β, snail, but negatively correlated with E-cadherin *in vivo* by IHC analysis (Fig. 6E and Fig. S6A-E). These data have further emphasized the positive correlation between Rab3D and EMT. Fig. 6F also showed that the level of plasma Hsp90α in Rab3D-MCF-7 xenografts was twice as much as that in the control group (n=5). Furthermore, stable Rab3D knockdown in the xenografts showed much lower level of Hsp90α in blood plasma compared with controls.

Taken together, our results support that Rab3D is necessary and sufficient to promote tumor metastasis. A working model for the effect of Rab3D on metastasis is shown in Fig. 6G.

## DISCUSSION

We have demonstrated the critical role of Rab3D in the regulation of tumor cell motility, invasion and metastasis. We have reported, for the first time, that the levels of Rab3D are elevated in clinical patient samples with many tumor types including breast, prostate, lung, colon, ovary, liver and skin cancers and are highly correlated with tumor TNM staging. Furthermore, the suppressions of Rab3D expression, which resulted in the deregulation of exosomes release and Hsp90α secretion, have repressed tumor cell motility and invasiveness. These findings support the notion that Rab3D is a relevant diagnostic and therapeutic target against tumor progression.

### Rab3D can serve as a promising biomarker for tumor progression

It has been reported that the patients with high Rab27A expression have a poor overall survival and is an independent prognostic marker for pancreatic ductal adenocarcinoma [37]. Moreover, the hepatocellular carcinoma patients with high level of Rab27A or Rab27B significantly reduce the overall survival [38]. In this study, the levels of Rab3D in the normal tissues or benign tumors are low, whereas the high levels of Rab3D are closely correlated with tumor TNM staging not grade, showing that Rab3D is a potential selective target for cancer progression. But we don't detect the correlation between Rab3D expression and the relative risk of death due to lack of clinical resources. Large scale, randomized, clinical trials are needed in the future to reveal that Rab3D is the valuable prognostic indicator for cancer patients in the clinic.

More importantly, the abnormal expression of Rab3D in cancer cells is not tissue- and tumor type-specific. The levels of Rab3D in breast, prostate, lung, colon, ovary, liver and skin carcinomas tissues are much higher than that in normal tissues with the IHC analysis, which suggests that Rab3D can be a broad-spectrum diagnostic biomarker for tumor progression in pathological examinations and refers the patients for diagnosis and treatment. Although 8 malignant cases had a Rab3D score of 0, there were 92 cases that were positive and all of the normal tissues had the score of 0, which indicated the good sensitivity and specificity of Rab3D as a diagnostic biomarker to detect carcinoma. On the other hand, our finding that the up-regulation of Rab3D protein levels in the majority of cancers appears inconsistent with the published data from Hendrix A and his co-workers, who reported that there was no difference of Rab3D in mRNA level in clinical breast cancer specimens [17]. But the inconsistence between mRNA and protein level changes implies that in the tumor cells there might be some important effectors to mediate the post-transcriptional regulation of Rab3D and its stabilization.

### Rab3D controls the exosomes release and Hsp90α secretion

Rab27A and Rab27B GTPases can regulate the release of exosomes, which is an important process for metastatic signaling transduction [15, 16]. In this study, we have found Rab3D as a new member to regulate exosomes. Rab3D can affect the total amount of exosomes but not their sizes. The roles of Rab3D have been described in Weibel-Palade body exocytosis, amylase release in exocrine cells and secretory vesicle traffic in lacrimal acinar cells [27, 31, 39]. But here, the association between Rab3D and exosomes release in tumor is uncovered for the first time. And exosomes have its roles in cancer development and metastasis [40]. Thus it is possible that Rab3D can promote tumor metastasis due to the secreted proteins from exosomes. Recently, large numbers of proteins in exosomes have been identified including adhesion molecules (e.g., integrin), membrane trafficking molecules (e.g., Rab proteins), chaperones (e.g., Hsp70 and Hsp90), signal transduction proteins (e.g., protein kinases), and immunosuppressive proteins (e.g., TGF-β) [41].

In this study, we focus on the important chaperone-Hsp90α, which has no signal peptide. Hsp90α is secreted *via* exosomes and strictly regulated by intracellular signaling pathways [42-44]. Secreted Hsp90α can stabilize and therefore activate the function of MMPs [25, 45, 46]. We have demonstrated that Rab3D is essential for Hsp90α secretion. More interestingly, after knocking down other secretion-related Rabs including Rab3B, Rab11A/B, Rab27A/B, Rab35 and Rab37, separately, knockdown of Rab3D resulted in the lowest Hsp90α expression, suggesting the significant role of Rab3D in Hsp90α secretion. Therefore, Hsp90α might be mainly sorted into Rab3D-exosomes and finally released out of cells. Post-translational modifications of Hsp90 contributions its function of chaperone [47] and the secretion of Hsp90α is regulated by its phosphorylation of Thr90 [24]. Therefore, whether post-translational modifications control the Hsp90α trafficking into exosomes, which deserves further study.

### Rab3D mediates EMT process and can be a novel option for anti-tumor metastasis

EMT is the key player during tumor metastasis, in which E-cadherin is down-regulated and N-cadherin is increased [48, 49]. Overexpression of Rab25 contributes to metastasis of bladder cancer through the induction of EMT [50]. We thus detected the EMT marker in Rab3D knockdown or overexpression tumor cells, and accordingly our data showed that Rab3D regulates EMT induction of tumor cells *via* the activation of Akt/GSK-3β/Snail pathway. One of the major reasons for Rab3D-mediated EMT is that the level of exosomes is increased. In fact, typical EMT hallmark proteins in cells have been found in exosomes, regulating the tumor microenvironment to promote the metastatic progression [51]. Furthermore, secreted Hsp90 has been reported as a driver of EMT events [52], which is consistent with our finding that secreted Hsp90 is essential for Rab3D-induced cancer cell migration and invasion. In this study, we believe Rab3D has its potential as a therapeutic target in cancer and can be a novel option to treat tumor metastasis. However, we also need to concern the side effects from a therapeutic perspective because Rab3D has its physiological role in granulocytes and alveolar epithelial cells [53].

In summary, our study provides an important theoretical foundation for us to understand the role of Rab3D in tumor progression and metastasis, suggesting that Rab3D is a potential diagnostic biomarker and a novel therapeutic target for anti-tumor metastasis. A better understanding of this biological function can lead to novel therapeutic strategies. In the future, we can develop small-molecule inhibitors of Rab3D *via* rational drug design for the treatment of cancer.

## MATERIALS AND METHODS

### Cell culture

The methods of cell culture were performed as previously described [24]. Breast cancer cells MCF-7, MDA-MB-231 and melanoma cells B16-F0, B16-F1, B16-F10 (ATCC) were maintained in DMEM supplemented with 10% fetal bovine serum, 100 U/ml penicillin, and 100 μg/ml streptomycin (Hyclone). Breast cancer cells SKBr-3 were cultured in RPMI 1640 medium supplemented with 10% fetal bovine serum, 100 U/ml penicillin, and 100 μg/ml streptomycin (Hyclone).

### Transfection

To construct the HA-tagged Rab3D plasmid, the primers 5′-CCCAAGCTTATGGCATCAGCTGGAGACA-3′ and 5′-CCGGAATTCCTAGCAGCTGCAGCTGCT-3′ were synthesized by Invitrogen. Its cDNA was cloned into the pcDNA3.0 vector and confirmed by sequencing. To generate GFP-tagged Rab3D plasmid, the primers CCCAAGCTTATGGCATCAGCTGGAGACA-3′ and CGCGGATCCCTAGCAGCTGCAGCTGCT-3′ were synthesized by Invitrogen. Its cDNA was cloned into the pEGFP-C2 vector and confirmed by sequencing. Mutant forms of Rab3D that encoded the N135I, T36N, and Q81L proteins were generated by polymerase chain reaction (PCR) site-directed mutagenesis. Transient overexpression of Rab3D was obtained by transfection with HA-tagged Rab3D plasmid using the Turbofect agent (Thermo). Stable overexpression of Rab3D was obtained by Lentiviral transfection. Lentiviral Rab3D vector was purchased from Genepharm.

### RNA interference

Down-regulation of Rab3D in MDA-MB-231 cells was obtained by transfection of its siRNA. The siRNAs for Rab3D were purchased from GenePharma. Scrambled siRNA was purchased from Gene-Pharma (Shanghai, China). The sequences of effective siRNA to repress Rab3D expression are listed as follows: Rab3D siRNA-1: 5′-GCAGCAGAUCAGAACUUCGACUAUA3′; Rab3D siRNA-2: 5′-CGAACGUGUUGUGCCUGCUGAGGAU-3′. The siRNAs were transfected with Lipofectamine 2000 reagent (Invitrogen) according to the manufacturer's protocol. Stable knockdown of Rab3D cells was generated by Lentiviral transfection of shRab3D (Genepharm). The sequence of effective shRNA for Rab3D is 5′-CGAACGUGUUGUGCCUGCUGAGGAU-3′. After 48 h transfection, Western Blot was applied to evaluate the knock-down efficiency.

### Western blot analysis

Western Blot analyses were performed as previously described [24]. Cells were harvested, denatured, and subjected to SDS-PAGE. Proteins were transferred to polyvinylidene difluoride (PVDF) membrane, immunoblotted with the appropriated primary antibodies overnight at 4 °C, incubated with horseradish peroxidase-conjugated secondary antibodies for 1 h at room temperature, then detected with an enhanced chemiluminescence system (Roche) according to the manufacturer's protocol.

### Immunofluorescence

The cells were fixed in 4% paraformaldehyde for 15 min at room temperature and blocked with 10% goat serum for 60 min at room temperature. Coverslips were then incubated at 4 °C overnight with the appropriate primary antibody (anti-Hsp90α antibody). TRITC labeled phalloidin was used to visualize actin cytoskeleton. Images were photographed by Nikon microscope.

### Immunohistochemistry (IHC)

Briefly, antigen retrieval was performed by heating for 15 min in 10 mmol/L sodium citrate buffer in a microwave oven. The paraffin-embedded tumor tissue slides were incubated with a primary antibody with an available dilution overnight at 4 °C.

### Tissue microarray

Breast cancer tissue microarray (BR1006), which contained 50 clinical female specimens (the age range is 19 - 82, including cancer adjacent normal breast tissue, fibro adenoma, cystosarcoma phyllodes, carcinosarcoma, medullary carcinoma, Paget's disease, mucinous adenocarcinoma and invasive ductal carcinoma), was purchased from Alenabio (Xi'an, China).

Cancer tissue microarray (MC246, Alenabio) contains 12 carcinomas (prostate, lung, colon, breast, ovary, uterine, esophagus, stomach, liver, kidney, skin and cerebrum) and their normal tissues near the cancer.

Breast cancer tissue microarray (BC081120) were purchased from Alenabio (Xi'an, China), that contained 110 clinical female specimens including cancer adjacent normal breast tissue and malignant tissues with different staging and grading.

Briefly, tissue sections were immunostained with anti-human Rab3D monoclonal antibody according to the protocol of immunofluorescent staining. The histochemical score is assessed by intensity of staining. For the intensity, the score index is 0 (negative), 1 (weak), 2 (moderate) and 3 (strong).

### Animal studies

All animal studies were approved by the Institutional Animal Care and Use Committee of Tsinghua University. Tumor cells stably expressing or knocking down Rab3D mixed in Matrigel 1:1 were injected into the mammary fat pad of each mouse, respectively (2 × 10^6^ cells per mouse). Tumor volume was estimated by using the equation, *V* = 0.5 × *a* × *b^2^*, *a* is the length of the major axis of the tumor, and *b* is the length of the minor axis.

For the metastasis analysis, lungs were extracted, weighed and fixed in 4% paraformaldehyde for 4 h, then paraffin embedded, sectioned, and applied to hematoxylin and eosin staining or IHC and Immunofluorescence assay for second tumor sections.

### Statistical methods

Experimental results are expressed as mean value ± SD. Statistically significant differences between groups were determined using a 2-tailed unpaired Student *t* test, where a *P* < 0.05 is considered significant. The Chi-square test (*x*^2^ test) was used to test for differences between categorical variables for the tissue microarray.

## SUPPLEMENTARY MATERIAL, TABLES AND FIGURES


