# Supplementary material for: High expression of small GTPase Rab3D promotes cancer progression and metastasis
Source: Oncotarget. 2015 Mar 14;6(13):11125–38. doi: 10.18632/oncotarget.3575 (PMC4484444; doi:10.18632/oncotarget.3575)
Supplement: Supplementary file 1 [file oncotarget-06-11125-s001.pdf]

High expression of small GTPase Rab3D promotes cancer progression and metastasis

Supplemental Material

Antibodies and Reagents

The primary antibodies used in this study were 1:4000 Rat anti-human Hsp90α mAb (Stressgen, Canada), 1:3000 Rabbit anti-Rab3D mAb (Epitomics), 1:1000 mouse anti-Alix mAb (Santa Cruz Biotechnology, USA), 1:2000 mouse anti-GAPDH mAb (ZSGB-BIO, China), 1:1000 Rabbit anti-CD63 pAb (Bioworld, China), 1:500 Rabbit anti-N-cadherin pAb (Bioworld, China), 1:500 Rabbit anti-E-cadherin pAb (Bioworld, China), 1:1000 Rabbit anti-Snail1 pAb (Bioworld, China), 1:1000 Rabbit anti-Twist pAb (Proteintech, USA) and 1:1000 Rabbit anti-ZEB1 pAb (Proteintech, USA). An anti-Hsp90α monoclonal neutralizing antibody, developed by our lab, used in *in vitro* studies and animal studies can cross react with human Hsp90α. Its specificity and efficiency were verified by Western Blot and ELISA.

Cell invasion assay

For the Matrigel invasion assay, 5×10^4 cells in serum-free culture medium were inoculated into the 6.5-mm millicell inserts (8 μm pore size, Millipore, USA) coated with Matrigel (BD, USA) on the upper side of the filter membranes. 1% serum was added in the culture medium in the lower chamber. Parallel repetition of experiment was at least twice. Then the cells were allowed to migrate through the inserts for 48 h. Cells at the lower surface of the filter membranes were stained with 1% eosin and counted by microscope in 5 random fields per insert (magnification, ×100).

Wound healing assays

Cells were grown on 6-well plates. The monolayer was disrupted with a cell scraper of 1 mm, and filmed at 0, 36 h in a phase contrast video microscope (Nikon ELWD 0.3) (magnification,

×100). Experiments were done in duplicate, and nine fields of each well were recorded. Relative migrated distance was calculated between the wound edges at different time points.

Cell proliferation assay

100 μL of control and Rab3D knockdown MDA-MB231 cells or Rab3D-overexpressing MCF-7 cells suspension (2× 10^4 /mL) were seeded on 96-well plates in DMEM containing 2 % FBS for 48 hours. 20 μL of CCK-8 (Dojindo) is added into each well and incubated for 2 hours. The absorption was detected at 450 nm.

Quantitative Real-time PCR

Total RNA was isolated using the Trizol reagent (Invitrogen) according to the manufacturer's protocol. First-strand cDNA was synthesized using Revert Aid First-strand cDNA synthesis kit (Thermo). Quantitative real-time PCR was performed using the comparative Cr method to detect relative gene expression.

Exosomes isolation

Cells were cultured in the medium without FBS (FBS, Hyclone). Exosomes were purified by sequential centrifugation steps (1). Supernatant fractions were centrifuged at 300 g for 10 min, 20,000 g for 30 min and then at 100,000 g for 120 min. The exosomes pellets were resuspended in PBS and collected by ultracentrifugation at 100,000 g for 120 min (Beckman).

Mass Spectrometry

The analyses were performed as previously described (2). Gel bands were digested by trypsin. Peptide mixture was analyzed by liquid chromatography–mass spectrometry (Agilent 6300 Series Ion Trap Liquid Chromatography/ Mass Systems). The MS data were searched against a subset of mouse proteins from the SWISS-PROT protein sequence database. Peptide confidence is high.

Electron microscopy

Exosomes were dropped onto a formvar-carbon–coated grid and fixed in 4% paraformaldehyde. After washing in PBS, the exosomes were washed in water and stained with saturated aqueous uranyl oxalate for 5 min. The excess liquid was then removed. The grid was dried at room temperature for 10 min and viewed at 20,000 and 50,000 magnification using an electron microscope (Hitachi).

Dynamic light scattering (DLS)

The mean hydrodynamic diameter of exosomes and size distribution was estimated by dynamic light scattering (DLS). The light scattering was recorded with 5 replicate measurements.

Table S1. Characteristics of benign and malignant breast cancer patients in tissue microarray assays

Table S2. Characteristics of 12 different cancer patient samples and cancer adjacent normal tissues

Table S3. Characteristics of 100 malignant breast cancer patient samples with define TNM and grade

Table S1. Tumor composition of 50 patients samples with clinic-pathological parameters

Number	Gender	Year	Organ	pathologic diagnosis	Grade	Type	Score
A1, A2	F	65	Breast	Invasive ductal carcinoma	1	Malignant	1
A3, A4	F	38	Breast	Invasive ductal carcinoma	2	Malignant	3
A5, A6	F	53	Breast	Invasive ductal carcinoma	2	Malignant	2
A7, A8	F	28	Breast	Invasive ductal carcinoma	2	Malignant	3
A9, A10	F	29	Breast	Invasive ductal carcinoma	1	Malignant	2
B1, B2	F	72	Breast	Invasive ductal carcinoma	2	Malignant	0
B3, B4	F	62	Breast	Invasive ductal carcinoma	2	Malignant	2
B5, B6	F	68	Breast	Invasive ductal carcinoma	2	Malignant	3
B7, B8	F	53	Breast	Mucinous adenocarcinoma	1	Malignant	1
B9, B10	F	66	Breast	Mucinous adenocarcinoma	1	Malignant	2
C1, C2	F	47	Breast	Mucinous adenocarcinoma	1	Malignant	1
C3, C4	F	65	Breast	Mucinous adenocarcinoma	2	Malignant	2
C5, C6	F	52	Breast	Mucinous adenocarcinoma	2	Malignant	2
C7, C8	F	41	Breast	Mucinous adenocarcinoma	2	Malignant	0
C9, C10	F	40	Breast	Mucinous adenocarcinoma	2-3	Malignant	2
D1, D2	F	45	Breast	Mucinous adenocarcinoma	2	Malignant	2
D3, D4	F	53	Breast	Medullary carcinoma	-	Malignant	2
D5, D6	F	60	Breast	Medullary carcinoma	-	Malignant	3
D7, D8	F	51	Breast	Medullary carcinoma	-	Malignant	2
D9, D10	F	31	Breast	Medullary carcinoma	-	Malignant	2
E1, E2	F	72	Breast	Medullary carcinoma	-	Malignant	2
E3, E4	F	55	Breast	Medullary carcinoma	-	Malignant	2
E5, E6	F	58	Breast	Paget's disease	-	Malignant	3
E7, E8	F	53	Breast	Paget's disease	-	Malignant	3
E9, E10	F	63	Breast	Paget's disease	-	Malignant	3
F1, F2	F	82	Breast	Paget's disease	-	Malignant	3
F3, F4	F	87	Breast	Carcinosarcoma	-	Malignant	3
F5, F6	F	39	Breast	Carcinosarcoma	-	Malignant	1
F7, F8	F	33	Breast	Cystosarcoma phyllodes	-	Malignant	3
F9, F10	F	48	Breast	Cystosarcoma phyllodes	-	Malignant	1
G1, G2	F	40	Breast	Cystosarcoma phyllodes	-	Malignant	0
G3, G4	F	32	Breast	Cystosarcoma phyllodes	-	Malignant	2
G5, G6	F	37	Breast	Cystosarcoma phyllodes	-	Malignant	3
G7, G8	F	69	Breast	Cystosarcoma phyllodes	-	Malignant	2
G9, G10	F	40	Breast	Cystosarcoma phyllodes	-	Malignant	0
H1, H2	F	40	Breast	Cystosarcoma phyllodes	-	Malignant	0
H3, H4	F	30	Breast	Fibroadenoma	-	Benign	1

H5, H6	F	23	Breast	Fibroadenoma	-	Benign	1
H7, H8	F	23	Breast	Fibroadenoma	-	Benign	1
H9, H10	F	32	Breast	Fibroadenoma	-	Benign	1
I1, I2	F	19	Breast	Fibroadenoma	-	Benign	1
I3, I4	F	42	Breast	Fibroadenoma	-	Benign	0
I5, I6	F	34	Breast	Fibroadenoma	-	Benign	0
I7, I8	F	46	Breast	Fibroadenoma	-	Benign	1
I9, I10	F	42	Breast	Cancer adjacent normal breast tissues	-	Normal	0
J1, J2	F	50	Breast	Cancer adjacent normal breast tissues	-	Normal	0
J3, J4	F	57	Breast	Cancer adjacent normal breast tissues	-	Normal	0
J5, J6	F	47	Breast	Cancer adjacent normal breast tissues	-	Normal	0
J7, J8	F	39	Breast	Cancer adjacent normal breast tissues	-	Normal	0
J9, J10	F	38	Breast	Cancer adjacent normal breast tissues	-	Normal	0

Table S2. Tumor composition of 12 patient samples with clinic-pathological parameters

Number	Gender	Year	Organ	pathologic diagnosis	Grade	Stage	Type
A1	M	71	Prostate	Adenocarcinoma	1	II	Malignant
A2	M	71	Prostate	Cancer adjacent normal prostate tissue	-	-	NAT
A3	M	55	Lung	Squamous cell carcinoma	2	II	Malignant
A4	M	55	Lung	Cancer adjacent normal lung tissue	-	-	NAT
A5	M	54	Colon	Adenocarcinoma	2	I	Malignant
A6	M	54	Colon	Cancer adjacent normal colon tissue	-		NAT
B1	F	40	Breast	Invasive ductal carcinoma	2	Ib	Malignant
B2	F	40	Breast	Cancer adjacent normal breast tissue	-	-	NAT
B3	F	68	Ovary	Serous adenocarcinoma	-	II	Malignant
B4	F	68	Ovary	Cancer adjacent normal ovary tissue	-	-	NAT
B5	F	50	Uterine cervix	Squamous cell carcinoma	3	I	Malignant
B6	F	50	Uterine cervix	Cancer adjacent normal cervical canals tissue	-		NAT
C1	F	49	Esophagus	Squamous cell carcinoma	1	IIa	Malignant
C2	F	49	Esophagus	Cancer adjacent normal esophagus tissue	-	-	NAT
C3	F	78	Stomach	Adenocarcinoma	1	II	Malignant
C4	F	78	Stomach	Cancer adjacent normal stomach tissue	-	-	NAT
C5	F	65	Liver	Hepatocellular carcinoma	2	II	Malignant
C6	F	65	Liver	Cancer adjacent normal hepatic tissue	-	-	NAT
D1	M	41	Kidney	Clear cell carcinoma	2	I	Malignant
D2	M	41	Kidney	Cancer adjacent normal kidney tissue	-	-	NAT
D3	M	52	Skin	Malignant melanoma of right heel	-	IV	Malignant
D4	M	52	Skin	Cancer adjacent normal skin tissue	-	-	NAT
D5	M	51	Cerebrum	Glioblastoma	4	-	Malignant

D6	M	51	Cerebrum	Cancer adjacent normal cerebrum tissue	-	-	NAT

Table S3. Tumor composition of 110 patients samples with clinic-pathological parameters

Number	Gender	Year	Organ	pathologic diagnosis	Grade	Stage	Score
A1	F	39	Breast	Invasive ductal carcinoma	1	IIa	1
A2	F	48	Breast	Invasive ductal carcinoma	1	IIIb	1
A3	F	43	Breast	Invasive ductal carcinoma	2	I	1
A4	F	45	Breast	Invasive ductal carcinoma	2	IIIb	2
A5	F	53	Breast	Invasive ductal carcinoma	1	IIa	3
A6	F	53	Breast	Invasive ductal carcinoma	2	IIb	0
A7	F	48	Breast	Invasive ductal carcinoma	1	IIIb	1
A8	F	37	Breast	Invasive ductal carcinoma	1	IIa	1
A9	F	32	Breast	Invasive ductal carcinoma	1	IIa	3
A10	F	32	Breast	Invasive ductal carcinoma	1	IIa	2
B1	F	41	Breast	Invasive ductal carcinoma	1	IIIa	1
B2	F	55	Breast	Invasive ductal carcinoma	1	IIa	2
B3	F	67	Breast	Invasive ductal carcinoma	1	IIa	1
B4	F	34	Breast	Invasive ductal carcinoma	1	IIb	2
B5	F	46	Breast	Invasive ductal carcinoma	2	I	0
B6	F	49	Breast	Invasive ductal carcinoma	1	IIa	1
B7	F	47	Breast	Invasive ductal carcinoma	1-2	I	0
B8	F	48	Breast	Invasive ductal carcinoma	1	IIIb	1
B9	F	52	Breast	Invasive ductal carcinoma	1	IIIa	2
B10	F	61	Breast	Invasive ductal carcinoma	1	IIIa	3
C1	F	38	Breast	Invasive ductal carcinoma	2	IIa	2
C2	F	45	Breast	Invasive ductal carcinoma	2	IIa	2
C3	F	38	Breast	Invasive ductal carcinoma	2	IIb	1
C4	F	63	Breast	Invasive ductal carcinoma	1	I	1
C5	F	32	Breast	Invasive ductal carcinoma	2	IIa	1
C6	F	45	Breast	Invasive ductal carcinoma	2	IIa	0
C7	F	54	Breast	Invasive ductal carcinoma	1	IIa	2
C8	F	47	Breast	Invasive ductal carcinoma	2	IIb	1
C9	F	51	Breast	Invasive ductal carcinoma	2	IIIa	3
C10	F	45	Breast	Invasive ductal carcinoma	2	I	3
D1	F	40	Breast	Invasive ductal carcinoma	2	I	1
D2	F	50	Breast	Invasive ductal carcinoma	2	IIa	2
D3	F	56	Breast	Invasive ductal carcinoma	2	IIa	3
D4	F	55	Breast	Invasive ductal carcinoma	2	IIa	3
D5	F	59	Breast	Invasive ductal carcinoma	2	IIa	1
D6	F	53	Breast	Invasive ductal carcinoma	2	IIb	1

D7	F	57	Breast	Invasive ductal carcinoma	2	IIa	2
D8	F	51	Breast	Invasive ductal carcinoma	2	IIb	1
D9	F	64	Breast	Invasive ductal carcinoma	2	IIa	2
D10	F	54	Breast	Invasive ductal carcinoma	2	I	2
E1	F	30	Breast	Invasive ductal carcinoma	2	I	2
E2	F	63	Breast	Invasive ductal carcinoma	2	IIa	1
E3	F	65	Breast	Invasive ductal carcinoma	2	IIa	1
E4	F	68	Breast	Invasive ductal carcinoma	2	IIa	2
E5	F	45	Breast	Invasive ductal carcinoma	2	IIIb	3
E6	F	58	Breast	Invasive ductal carcinoma	2	IIa	0
E7	F	42	Breast	Invasive ductal carcinoma	-	IIa	1
E8	F	40	Breast	Invasive ductal carcinoma	2	IIa	1
E9	F	42	Breast	Invasive ductal carcinoma	2	IIa	2
E10	F	46	Breast	Invasive ductal carcinoma	2	IIa	1
F1	F	39	Breast	Invasive ductal carcinoma	2	IIIb	3
F2	F	46	Breast	Invasive ductal carcinoma	-	IIa	0
F3	F	46	Breast	Invasive ductal carcinoma	2	IIa	2
F4	F	46	Breast	Invasive ductal carcinoma	2	IIIb	3
F5	F	39	Breast	Invasive ductal carcinoma	2	IIa	2
F6	F	46	Breast	Invasive ductal carcinoma	2	IIb	2
F7	F	32	Breast	Invasive ductal carcinoma	2	IIb	1
F8	F	48	Breast	Invasive ductal carcinoma	2	IIa	1
F9	F	46	Breast	Invasive ductal carcinoma	2	IIIb	1
F10	F	47	Breast	Invasive ductal carcinoma	2	IIb	2
G1	F	50	Breast	Invasive ductal carcinoma	2	IIa	1
G2	F	43	Breast	Invasive ductal carcinoma	2	I	2
G3	F	48	Breast	Invasive ductal carcinoma	2	IIa	1
G4	F	49	Breast	Invasive ductal carcinoma	2	IIa	2
G5	F	29	Breast	Invasive ductal carcinoma	2	IIa	3
G6	F	40	Breast	Invasive ductal carcinoma	-	IIIb	3
G7	F	38	Breast	Invasive ductal carcinoma	1	I	1
G8	F	38	Breast	Invasive ductal carcinoma	2	IIb	3
G9	F	62	Breast	Invasive ductal carcinoma	2	IIa	2
G10	F	44	Breast	Invasive ductal carcinoma	1	I	1
H1	F	68	Breast	Invasive ductal carcinoma	2	IIa	2
H2	F	40	Breast	Invasive ductal carcinoma	1	IIb	3
H3	F	53	Breast	Invasive ductal carcinoma	2	IIa	2
H4	F	63	Breast	Invasive ductal carcinoma	2	IIa	2
H5	F	48	Breast	Invasive ductal carcinoma	2	IIIb	3

H6	F	62	Breast	Invasive ductal carcinoma	2	IIb	3
H7	F	46	Breast	Invasive ductal carcinoma	2	I	1
H8	F	50	Breast	Invasive ductal carcinoma	2	IIa	1
H9	F	39	Breast	Invasive ductal carcinoma	2	IIa	1
H10	F	60	Breast	Invasive ductal carcinoma	2	IIIb	3
I1	F	41	Breast	Invasive ductal carcinoma	2	IIIb	2
I2	F	37	Breast	Invasive ductal carcinoma	2	IIb	1
I3	F	40	Breast	Invasive ductal carcinoma	2	IIa	2
I4	F	49	Breast	Invasive ductal carcinoma	2	IIa	1
I5	F	50	Breast	Invasive ductal carcinoma	-	IIb	0
I6	F	48	Breast	Invasive ductal carcinoma	2	I	1
I7	F	74	Breast	Invasive ductal carcinoma	2	IIa	2
I8	F	74	Breast	Invasive ductal carcinoma	-	IIa	0
I9	F	40	Breast	Invasive ductal carcinoma	2	IV	3
I10	F	70	Breast	Invasive ductal carcinoma	2	IIa	1
J1	F	64	Breast	Invasive ductal carcinoma	-	IIa	1
J2	F	52	Breast	Invasive ductal carcinoma	2-3	IIb	2
J3	F	35	Breast	Invasive ductal carcinoma	2-3	IIa	2
J4	F	53	Breast	Invasive ductal carcinoma	2	IIa	2
J5	F	52	Breast	Invasive ductal carcinoma	3	I	2
J6	F	41	Breast	Invasive ductal carcinoma	3	IIa	2
J7	F	56	Breast	Invasive ductal carcinoma	2	IIa	2
J8	F	46	Breast	Invasive ductal carcinoma	3	IIIb	3
J9	F	44	Breast	Invasive ductal carcinoma	3	IIa	1
J10	F	38	Breast	Invasive ductal carcinoma	3	IIa	1
K1	F	43	Breast	Cancer adjacent normal breast tissue	-	-	0
K2	F	42	Breast	Cancer adjacent normal breast tissue	-	-	0
K3	F	47	Breast	Cancer adjacent normal breast tissue	-	-	0
K4	F	39	Breast	Cancer adjacent normal breast tissue	-	-	0
K5	F	49	Breast	Cancer adjacent normal breast tissue	-	-	0
K6	F	53	Breast	Cancer adjacent normal breast tissue	-	-	0
K7	F	51	Breast	Cancer adjacent normal breast tissue	-	-	0
K8	F	18	Breast	Cancer adjacent normal breast tissue	-	-	0
K9	F	44	Breast	Cancer adjacent normal breast tissue	-	-	0
K10	F	39	Breast	Cancer adjacent normal breast tissue	-	-	0

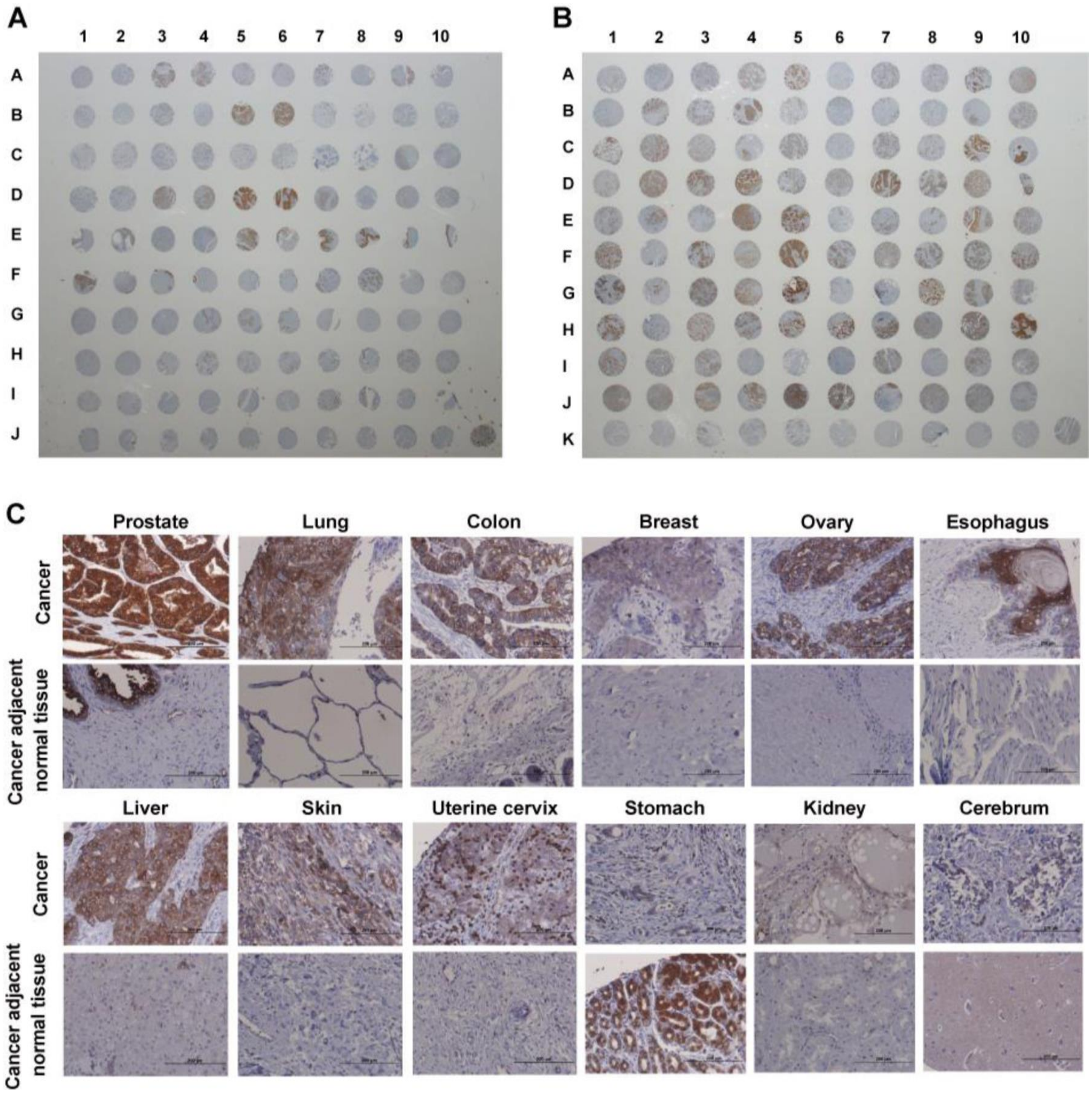

Fig. S1: Rab3D staining in different malignant cancer samples

(A-B) Related to Figure 1E and 1G. Overview of the immunohistochemistry results. Numbers up the microarrays and capital letters at the left can locate each tissue point. (C). Rab3D staining in 12 carcinomas (prostate, lung, colon, breast, ovary, uterine, esophagus, stomach, liver, kidney, skin and cerebrum) and their normal tissues near the cancer by IHC assay.

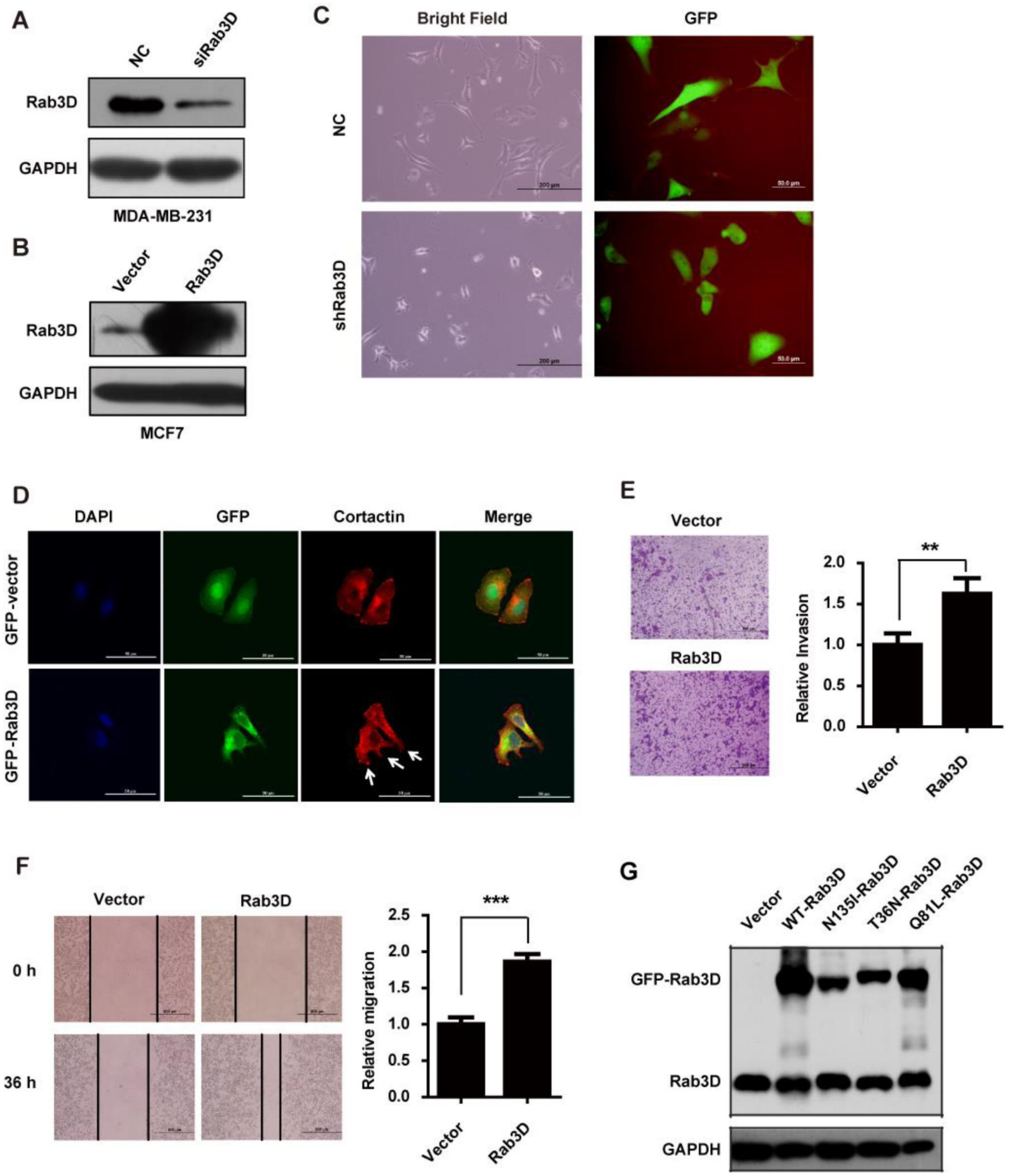

Fig. S2: The role of Rab3D in tumor cell motility

(A-B). Transfection efficiency. MCF-7 cells were transfected with GFP-control and GFP-Rab3D plasmid. MDA-MB-231 cells were transfected with scramble RNA and Rab3D siRNA,

separately. (C). The cell morphology in GFP-labeled shRab3D MDA-MB-231 cells. Scale bar, 50 μm. (D). The effect of overexpressing Rab3D on tumor cell morphology. MCF-7 cells were transfected with GFP-Rab3D-expressing plasmid and spreading protrusions marker cortactin was stained with Dy3 labeled second antibody. (E). Over-expressing Rab3D in MCF-7 resulted in dramatically increased cell motility (migration). Representative images and quantification of migration assay by MCF-7 cells transfected to express Rab3D. Scale bar, 200 μm. ** $p < 0.01$. (F). Over-expressing Rab3D in MCF-7 resulted in dramatically increased cell invasion. Representative images and quantification of the invasive phenotype induced by Rab3D MCF-7 cells. Scale bar, 100 μm. *** $p < 0.001$. (G). Western Blot analysis of Rab3D mutants.

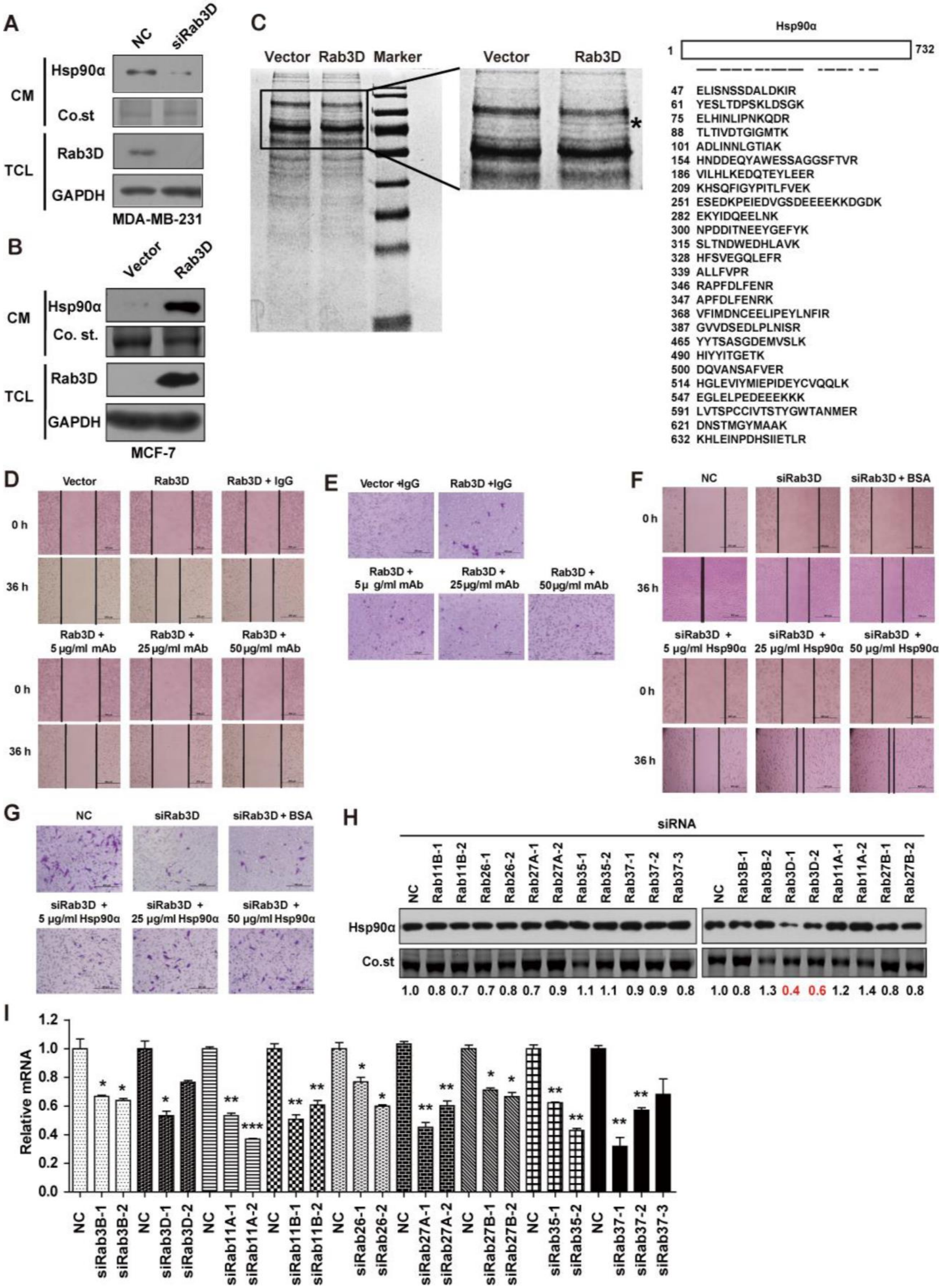
A
NC
siRab3D
CM
Hsp90α
Co.st
TCL
Rab3D
GAPDH
MDA-MB-231
B
Vector
Rab3D
CM
Hsp90α
Co. st.
TCL
Rab3D
GAPDH
MCF-7
C
Vector
Rab3D
Marker
Vector
Rab3D
*
Hsp90α
1
732
47 ELISNSSDALDKIR
61 YESLTDPSKLDSGK
75 ELHINLIPNKQDR
88 TLTIVDTGIGMTK
101 ADLINNLGTIAK
154 HNDDEQYAWESSAGGSFTVR
186 VILHLKEDQTEYLEER
209 KHSQFIGYPITLFVEK
251 ESEDKPEIEDVGSDEEEEKKDGDK
282 EKYIDQEELNK
300 NPDDITNEEYGEFYK
315 SLTNDWEDHLAVK
328 HFSVEGQLEFR
339 ALLFVPR
346 RAPFDLFENR
347 APFDLFENRK
368 VFIMDNCEELIPEYLNFIR
387 GVVDSEDLPLNISR
465 YYTSASGDEMVSLK
490 HIYYITGETK
500 DQVANSAFVER
514 HGLEVIYMIEPIDEYCVQQLK
547 EGLELPEDEEEKKK
591 LVTSPCCIVTSTYGWTANMER
621 DNSTMGYMAAK
632 KHLEINPDHSIIETLR
D
Vector
Rab3D
Rab3D + IgG
0 h
36 h
Rab3D + 5 μg/ml mAb
Rab3D + 25 μg/ml mAb
Rab3D + 50 μg/ml mAb
0 h
36 h
E
Vector +IgG
Rab3D +IgG
Rab3D + 5μ g/ml mAb
Rab3D + 25μg/ml mAb
Rab3D + 50μg/ml mAb
F
NC
siRab3D
siRab3D + BSA
0 h
36 h
siRab3D + 5 μg/ml Hsp90α
siRab3D + 25 μg/ml Hsp90α
siRab3D + 50 μg/ml Hsp90α
0 h
36 h
G
NC
siRab3D
siRab3D + BSA
siRab3D + 5 μg/ml Hsp90α
siRab3D + 25 μg/ml Hsp90α
siRab3D + 50 μg/ml Hsp90α
H
siRNA
NC
Rab11B-1
Rab11B-2
Rab26-1
Rab26-2
Rab27A-1
Rab27A-2
Rab35-1
Rab35-2
Rab37-1
Rab37-2
Rab37-3
NC
Rab3B-1
Rab3B-2
Rab3D-1
Rab3D-2
Rab11A-1
Rab11A-2
Rab27B-1
Rab27B-2
Hsp90α
Co.st
1.0 0.8 0.7 0.7 0.8 0.7 0.9 1.1 1.1 0.9 0.9 0.8
1.0 0.8 1.3 0.4 0.6 1.2 1.4 0.8 0.8
I
Relative mRNA
1.2
1.0
0.8
0.6
0.4
0.2
0.0
NC
siRab3B-1
siRab3B-2
NC
siRab3D-1
siRab3D-2
NC
siRab11A-1
siRab11A-2
NC
siRab11B-1
siRab11B-2
NC
siRab26-1
siRab26-2
NC
siRab27A-1
siRab27A-2
NC
siRab27B-1
siRab27B-2
NC
siRab35-1
siRab35-2
NC
siRab37-1
siRab37-2
siRab37-3

Fig. S3. Effect of exosome-related Rabs on Hsp90α secretion

(A-B). Western Blot analysis of Hsp90α in CM of MDA-MB-231 cells transfected with scramble RNA or Rab3D siRNA. (C). Mass spectrometry analysis identified Hsp90α and unique peptides were indicated. (D). Representative images of migration assay. The effect of recombinant Hsp90α on the migration of MDA-MB-231 transfected with scramble RNA or Rab3D siRNA. Scale bar, 200 μm. (E). Representative images of invasion in siRab3D MDA-MB-231 with or without addition of recombinant Hsp90α. Scale bar, 200 μm. (F). Representative images of migration assay. Rab3D-MCF-7 cells were treated with or without Hsp90α neutralizing antibody. Scale bar, 200 μm. (G). Representative images of matrigel invasion assay. The effect of blocking extracellular Hsp90α in Rab3D-MCF-7 cells. Scale bar, 200 μm. (H). MCF-7 cells were transfected with exosome-related Rabs siRNA including Rab3B, Rab3D, Rab11A, Rab11B, Rab27A, Rab27B, Rab35 and Rab37 separately. After 48h, the level of secreted Hsp90α in the CM was measured by Western Blot assay. (I). mRNA level of Rabs in these cells by real-time qRT-PCR analysis. Statistically significant *P* values are indicated. . * $p < 0.05$, ** $p < 0.01$, *** $p < 0.001$.

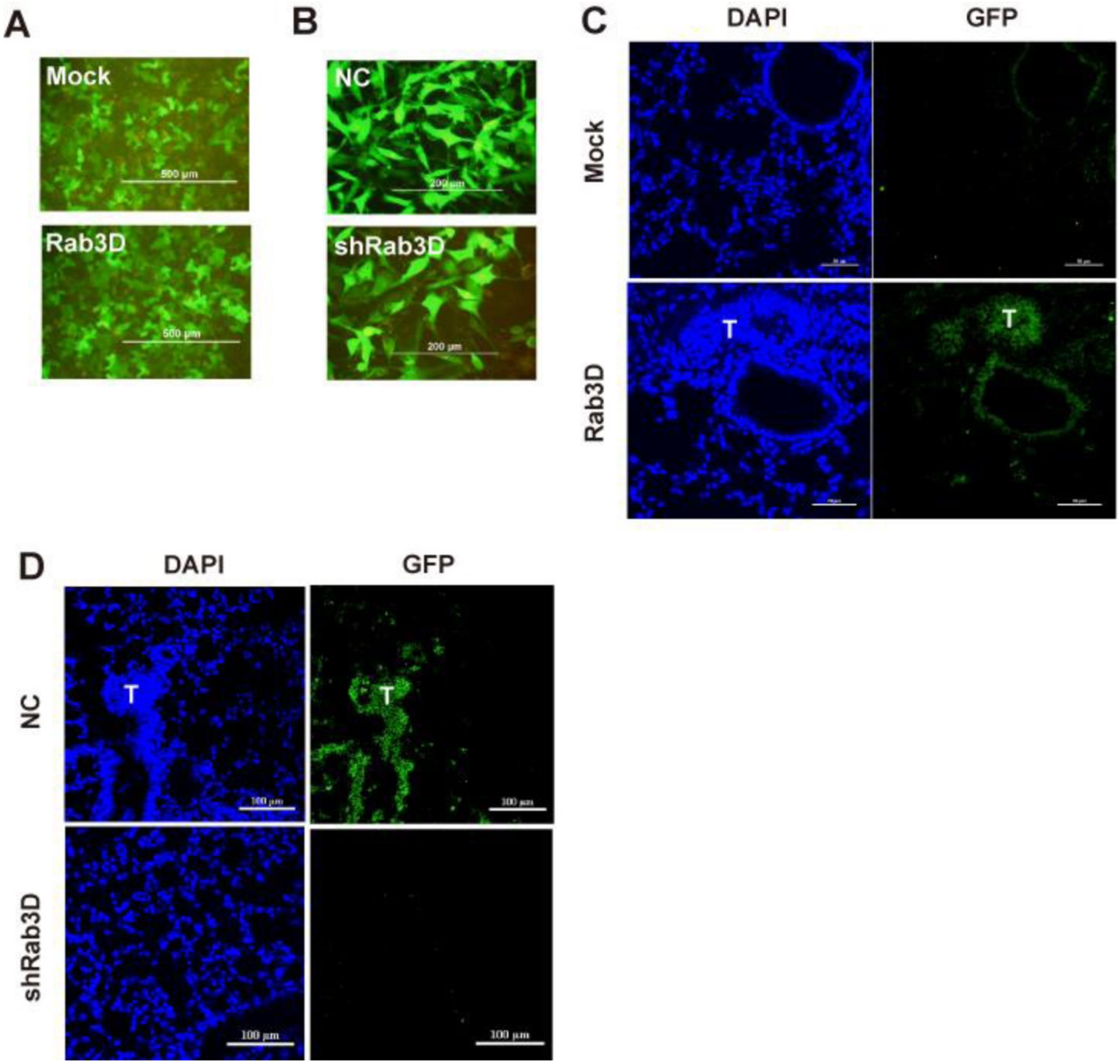

Fig. S4. The effect of GFP-Rab3D on tumor lung metastasis

(A-B). Images of stably transfected tumor cell lines. (C). Immunofluorescence images of lung in control and Rab3D-MCF-7 xenografts bearing mice. (D). Immunofluorescence images of lung in control and shRab3D-MDA-MB-231 xenografts bearing mice.

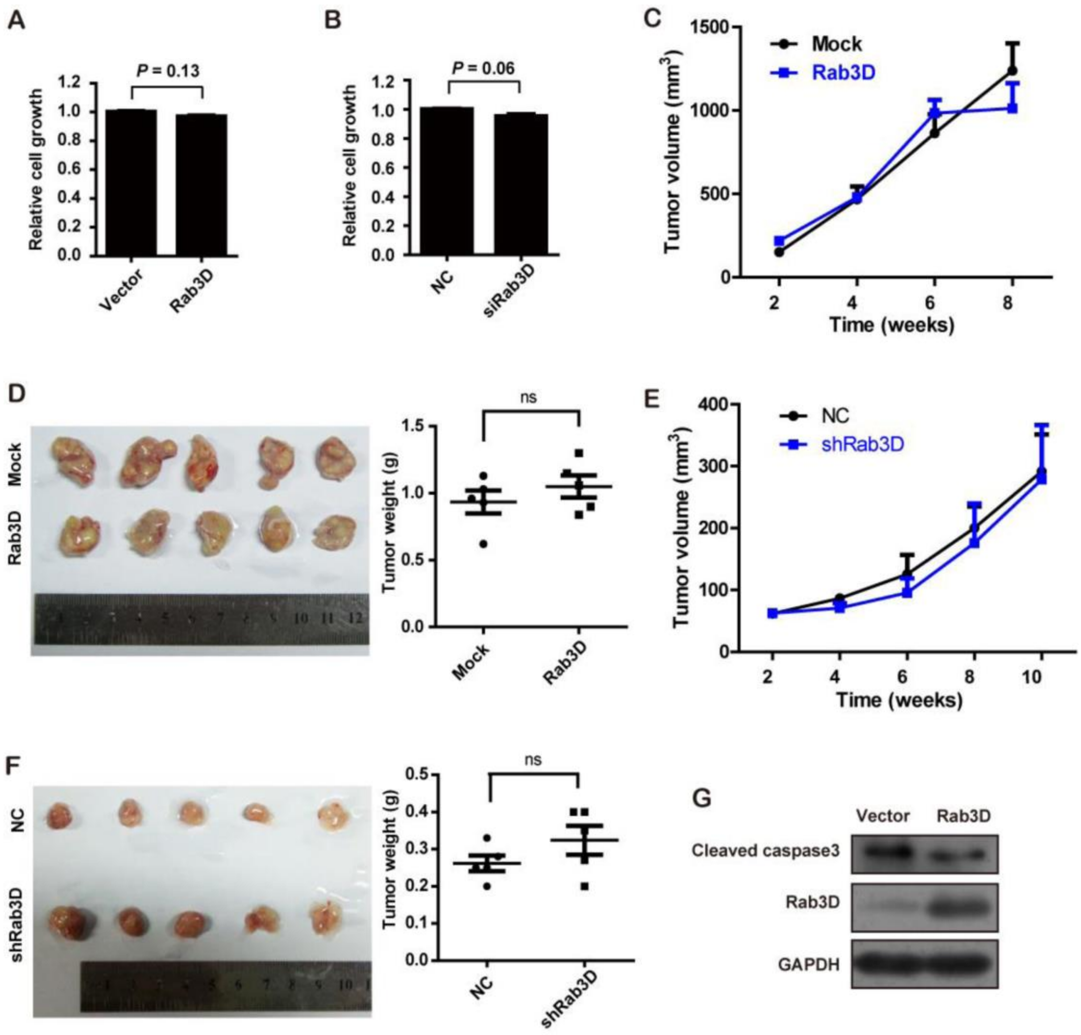

Fig. S5. ***In vitro*** **and in** ***vivo*** **tumor growth**

(A-B). Cell proliferation of siRab3D MDA-MB-231 cells and MCF-7 cells transiently expressed Rab3D. A total of 2000 cells were plated into each well of 96 wells plate for 2 days, and subsequently CCK-8 solution was added and incubated for 2 h. OD value at 450 nm was measured. Statistically significant p values are indicated. (C). Mean tumor volume in nude mice bearing MCF-GFP-Rab3D xenografts (n = 5 mice per group) assessed once every two weeks for 8 weeks. (D). Images of primary tumors (Left) and tumor weight (Right) in MCF-GFP-Rab3D and control group, ns means no significance. (E). Mean tumor volume in nude mice bearing

MDA-MB-231-GFP-shRab3D xenografts (*n* = 5 mice per group) assessed once every two weeks for 10 weeks. (F). Images of primary tumors (Left) and tumor weight (Right) in MDA-MB-231-GFP-shRab3D and control group. (G). The effect of Rab3D on tumor cell apoptosis.

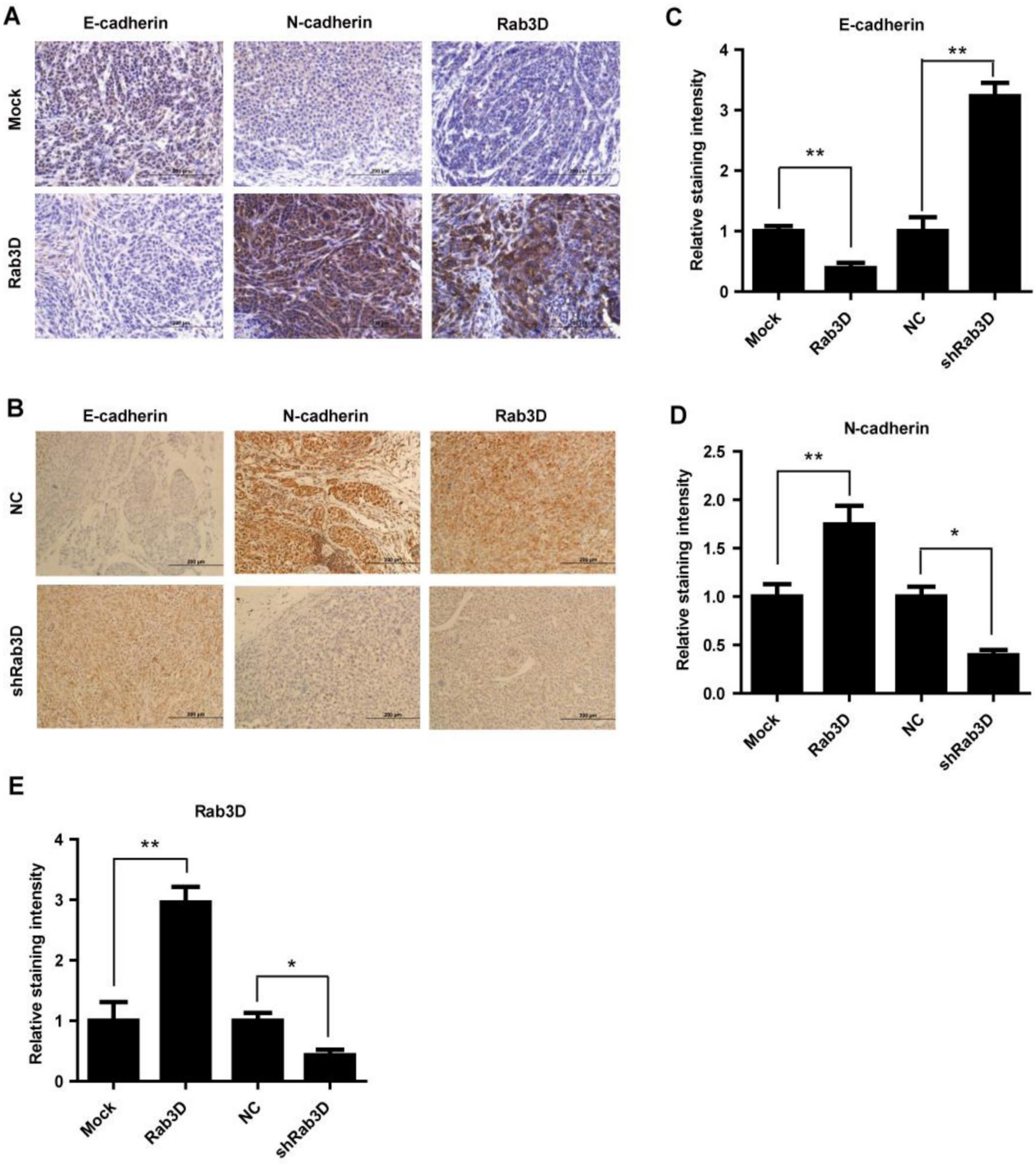

Fig. S6: Promotion of Rab3D in EMT process

(A-B). Immunohistochemical staining of E-cadherin or N-cadherin (EMT markers) in xenografts. Scale bar, 50μm. (C-E). Quantification of Immunohistochemical staining intensity. * $p < 0.05$, ** $p < 0.01$.

Referrences

1. Gross JC, Chaudhary V, Bartscherer K, Boutros M. Active Wnt proteins are secreted on exosomes. Nat Cell Biol. 2012;14:1036-45.
2. Lu XA, Wang X, Zhuo W, Jia L, Jiang Y, Fu Y, et al. The regulatory mechanism of a client kinase controlling its own release from Hsp90 chaperone machinery through phosphorylation. Biochem J. 2014;457:171-83.
